# Is it time to reconsider the administration of thiamine alone or in combination with vitamin C in critically ill patients? A meta-analysis of clinical trial studies

**DOI:** 10.1186/s40560-022-00594-8

**Published:** 2022-02-17

**Authors:** Nafiseh Shokri-mashhadi, Ali Aliyari, Zahra Hajhashemy, Saeed Saadat, Mohammad Hossein Rouhani

**Affiliations:** 1 Food Security Research Center and Department of Clinical Nutrition, School of Nutrition and Food Science, Isfahan University of Medical Sciences, Isfahan, Iran; 2 Food Security Research Center and Department of Community Nutrition, School of Nutrition and Food Science, Isfahan University of Medical Sciences, Isfahan, Iran; 3 Faculty of Mathematics and Natural Sciences, Department of Computer Sciences, Heinrich Heine Universität, Düsseldorf, Germany

**Keywords:** Intensive care unit, Thiamine, Vitamin C, Low-dose hydrocortisone, Meta-analysis

## Abstract

**Background:**

Although the effect of thiamine alone or in combination with vitamin C has been studied in multiple trials (RCT and interventional studies), their results are inconsistent. This meta-analysis aimed to assess impact of thiamine administration alone, thiamine in combination with vitamin C, and co-administration of low-dose hydrocortisone, vitamin C and thiamine (HVT) on clinical outcomes in critically ill patients.

**Methods and materials:**

After electronic searches on PubMed, Scopus, Cochrane Library, and Web of Science databases, initially 3367 papers were found, and 20 interventional studies were included in our analysis. We assessed the risk-difference between treatment and control (standard treatment) groups by pooling available data on ICU length of stay, number of ventilator free days, mortality, and changes in Sequential Organ Failure Assessment (SOFA) scores.

**Results:**

The results of present studies revealed no significant effect of thiamine in combination with vitamin C, and HVT on number of free days of ventilation. Thiamine alone supplementation was associated with high mortality percentage (WMD: 5.17%; 95% CI: 2.67, 7.67). Thiamine in combination with vitamin C had no significant impact on mortality rate. In contrast, HVT could decrease mortality rate (WMD: − 7.23%; 95% CI: − 10.31, − 4.16; I-square: 0.0%). There was no significant effect of thiamine alone, co-administration of thiamine and vitamin C, and HVT on ICU length of stay. The results of the meta-analysis showed that thiamine alone and HVT supplementation had no significant effect on SOFA score. Interestingly, co-supplementation of thiamine and vitamin C had a significant decreasing effect on SOFA score (WMD: − 0.73; 95% CI: − 1.29, − 0.17; I-square: 0.0%).

**Conclusion:**

In contrast to HVT, thiamine supplementation alone was associated with increased mortality rate in ICU. However, co-supplementation of thiamine and vitamin C had a significant decreasing effect on SOFA score.

## Highlights

- Thiamine, co-supplementation of thiamine and vitamin C and co-administration of hydrocortisone, vitamin C and thiamine (HVT) had no significant effect on free days of ventilation and ICU length of stay.
- HVT could decrease and thiamine supplementation could increase mortality rate in ICU.
- Co-supplementation of thiamine and vitamin C could decrease SOFA score.

## Introduction

Evidence supports the essential role of thiamine, a water-soluble vitamin, as an essential cofactor in maintenance of normal metabolism and central nervous function [1, 2]. Thiamine deficiency can lead to severe complications, including elevated lactate levels, cardiac failure, and brain dysfunction, which are linked to poor clinical outcomes and finally result in organ failure and mortality [3, 4].

It is reported that circulating thiamine levels in critically ill patients decrease over time, in particular in patients with diabetic ketoacidosis, chronic diarrhea, receiving chronic diuretic therapy, and the predominantly carbohydrate-based diet [5, 6]. Moreover, malnutrition is a common difficulty in the majority of patients admitted to hospital, especially in the intensive care unit (ICU), that could be potentially associated with an insufficient vital nutrients intake [7, 8]. In this concern, recent trials have focused on the efficacy of thiamine administration alone or in the combination with vitamin C in critically ill patients to achieve the best clinical outcomes for these patients. The results of some studies indicated that administration of thiamine alone could improve oxygen consumption in cardiac surgery patients [9], and be effective for prevention of postoperative delirium [10]. Moreover, it has beneficial effects in patients with burns and multiple trauma [11]. Nevertheless, the beneficial impact of thiamine supplementation did not confirm in the other studies [12, 13]. In this regard, previous published systematic review and meta-analysis investigated the effectiveness of vitamin C, thiamine, or co-administration of low-dose hydrocortisone, vitamin C and thiamine (HVT) on clinical outcomes in critically ill patients. However, the impact of treatment with thiamine alone has been not considered in mentioned studies [14–16]. Furthermore, there were no subgroup analyses based on the use of low-dose hydrocortisone or placebo in the control groups that it can affect the quality of results [14, 15]. It seems that pooled subgroup analyses from trials focused on administration of thiamine, may help clear information around thiamine supplementation for critically ill patients [17].

The main purpose of this review was to assess the effect of thiamine, co-supplementation of thiamine and vitamin C, and HVT on the clinical outcomes of adult critically ill patients.

## Materials and methods

### Literature research and data extraction

Current systematic review was carried out in compliance with the PRISMA statement [18]. Two investigators (N.Sh-m and A.Aliyari) independently conducted an electronic literature search using PubMed, Scopus, Cochrane Library, and Web of Science databases without any restrictions on language or date to identify the effect of thiamine alone or in conjunction with vitamin C and/or low-dose hydrocortisone when compared to standard care or placebo (From inception to November 2020). The electronic search strategy was done using the following keywords: ("Critical Care" OR "Critical Illness" OR "critical ill" OR "Intensive Care Units” OR “intensive care”) AND (thiamin* OR thiamine OR vitamin B1). The references pointed in the retrieved articles were also searched manually. In addition, similar queries were used for controlled vocabulary search [Mesh] at the same time.

All studies, which assessed the effect of administration of thiamine alone or in combination with vitamin C or/and low-dose hydrocortisone versus non-thiamine on patients admitted to ICU were included in this review. The exclusion criteria were as follows: (1) duplicated publications; (2) pediatric studies; (3) observational studies; (4) studies involving other supplements or treatments; (5) lacking the data of predefined endpoints; (6) animal models or in vivo studies; (7) review and meta-analysis studies, chapter's book, and conference papers; and (8) retrospective clinical trial. Patient population consisted of critically-ill adult patients (≥ 18 years). Following outcomes were considered in the present study: (1) SOFA score: It is a simple and objective score that allows for calculation of both the number and the severity of organ dysfunction in six different organs (respiratory, coagulatory, liver, cardiovascular, renal, and neurologic) [19]; (2) Length of stay in the ICU: It is a term introduced by the National Health Service (NHS) as the length of an inpatient hospitalization in ICU, calculated from the day when patients were admitted to the ICU to day of discharge based on the number of overnight stays [20]; (3) Ventilator-free days: Ventilator-free days at 28 days is an outcome that depends on organ dysfunction. It is defined in a 28 day period in critically ill patients and measured by the number of free days from mechanical ventilation to evaluate the efficacy of interventions [21]; and Mortality. Following the assessment of titles and abstracts, all identified studies were acquired as full-text. A third reviewer resolved any disagreements by discussion (Z.H).

We extracted the following information: author’s name, publication year, participant characteristics (sample size, gender, and age), medical conditions, intervention (type of compounds, dose, and duration) and main outcomes, including mortality, Sequential Organ Failure Assessment (SOFA) score, ICU length of stay, number of ventilator free days, and ICU mortality. The data were extracted independently by two investigators. We also contacted the corresponding author to obtain the data when necessary (N.Sh-M).

### Assessment of bias

In current meta-analysis, the quality assessment of included studies was performed through the use of Cochrane quality assessment tool [22]. Considering the Cochrane checklist, risk of bias for each study was examine based on 7 items including random sequence generation, allocation concealment, reporting bias, performance bias, detection bias, attrition bias, and other sources of bias. Each item was given “high risk” (  ), “low risk” (  ) or “unclear risk” (  ) score. “High risk” for studies with methodological defect that might affect their results; “low risk” for studies with no methodological defect in that item; and “unclear risk” for studies with insufficient information to examine the impact of method on findings. We considered the overall risk of bias for an article: low for studies that obtained “low risk” score in all domains. Moderate for studies with one or more “unclear risk” score, and high for studies with one or more “high risk” score (Table 1).

**Table 1:**
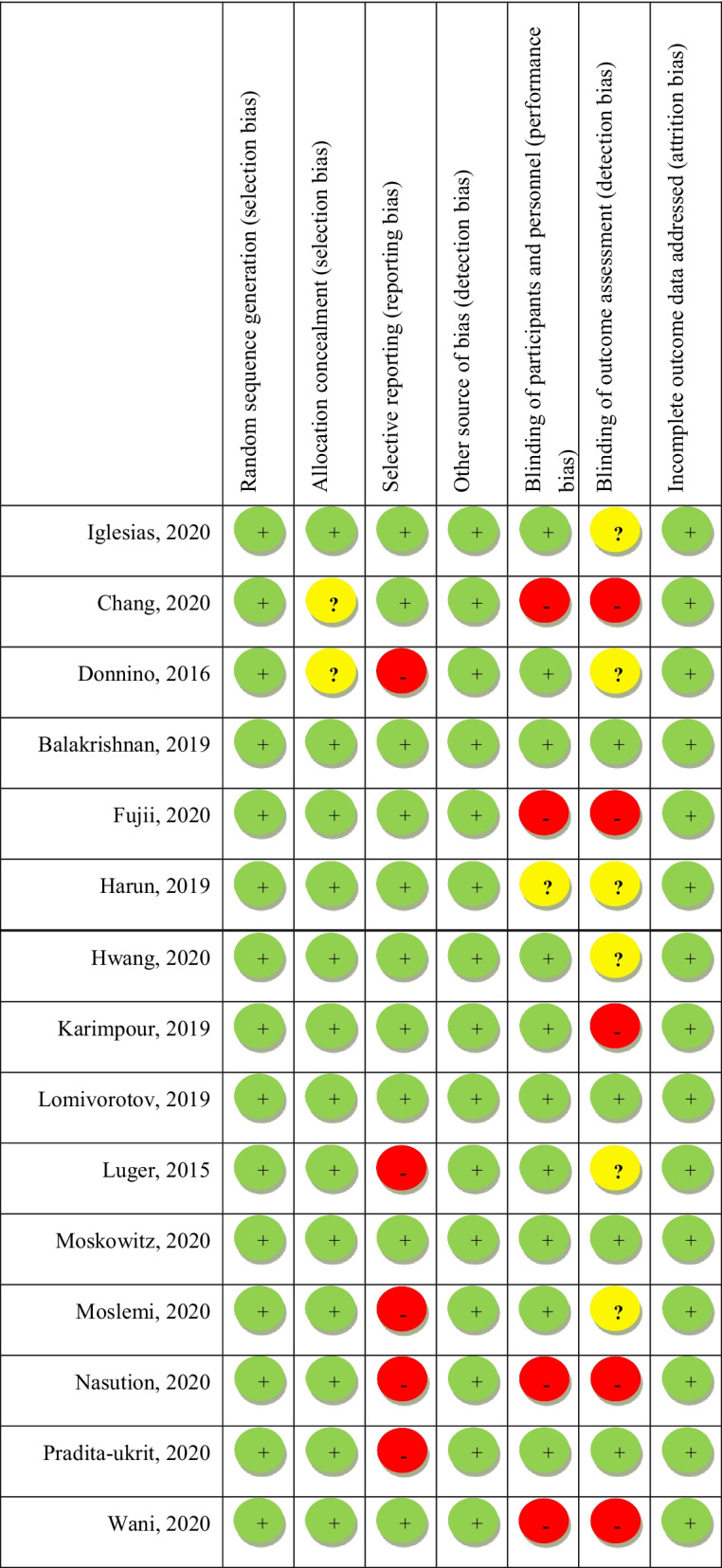
Quality assessment of included studies in the meta-analysis

### Statistical analysis

The effect of thiamine alone, co-supplementation of thiamine and vitamin C and/or HVT on clinical outcomes were evaluated by assessing the risk-difference between treatment and control (standard treatment) groups by pooling available data on mortality, ICU length of stay, change of SOFA score, and number of ventilator free days. Values are expressed as mean ± SD. In the cases that the risk-difference between treatment and control was reported as standard error (SE), we converted values to standard deviation (SD) using the formula SD = SE × √N. Mortality was reported as percent change in included studies. All outcomes were reported in a same values through the studies. The weighted mean difference was calculated by pooling effect sizes via a random-effects mode. Cutoffs for confidence intervals and definitions for both statistical significance (*p* < 0.05) and between-study heterogeneity (*I*^2^ > 50%) was considered. Robustness of the overall effect sizes was tested by sensitivity analysis Begg's rank correlation test and Egger's linear regression test were run to evaluate publication bias. Statistical analyses were performed using Stata software (version 11.2, Stata Corporation, College Station, Texas, USA).

## Results

### Study characteristics

**Fig. 1:**
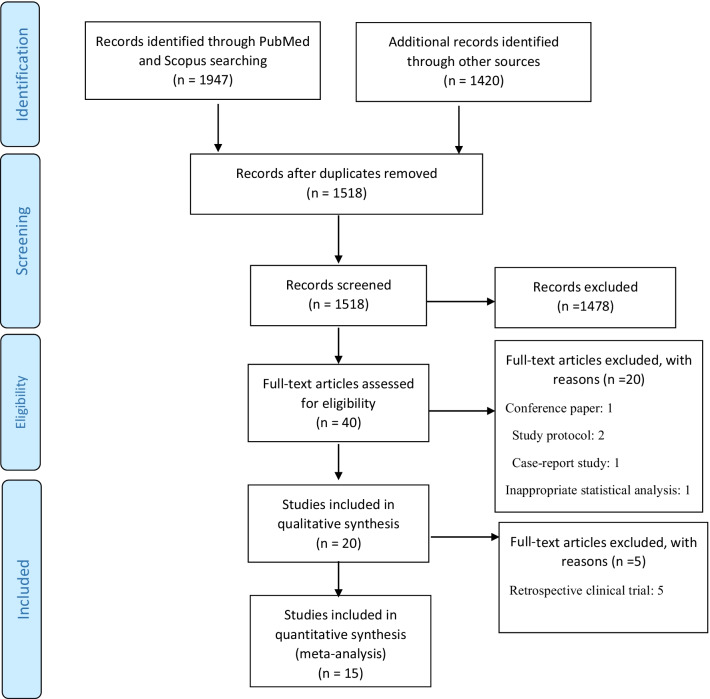
PRISMA flowchart of study selection process for effect of vitamin B1 on clinical outcomes of ICU patients

According to the search strategy, 3367 papers were initially found. After removing 1849 duplicates and 1478 unappropriated topic papers, 1518 studies were screened by title and abstract. The remaining 40 studies were considered to be of relevance, and full papers were carefully screened. In the following, 1 conference paper, 2 study protocol, 1 case-report study, 1 inappropriate statistical analysis, and 15 having no specified mentioned data were rejected. Therefore, 20 articles were included in the systematic review. Finally, we excluded 5 studies due to retrospective clinical trial design, and just 15 clinical trial articles enrolled in the meta-analysis [10, 12, 13, 23–34]. The selection process of papers summarized in Fig. 1.

Among 20 articles, fifteen studies were done in single center, and two trials conducted in two centers. Studies were done in different countries, including the USA [1, 8, 9, 11, 12, 23], Australia [14, 16], Korea [17, 18], Iran [2, 7], India [4, 10], Japan [3], China [5], Indonesia [13], Malaysia [15], and Thailand [22]. In addition, one of them has simultaneously done in Brazil, Australia, and New Zealand [6]. Fourteen studies performed on patients with same condition (sepsis), one study on patients with Acute respiratory distress syndrome (ARDS), one study on patients with cardiac arrest condition, and four studies on patients undergoing surgery (coronary artery bypass cardiac, and gastrointestinal surgery). Approximately 58% of all patients were male. The mean ages of participants ranged from 54 to 72 years, but one study recruiting patients with a mean age of 42 years [34]. Thiamine was administered in various dosing in the studies, ranging from 0.2 g (g) daily to 0.6. The dosage of administration of vitamin C with thiamine was ranged from 2 g/day to 6, and the duration of supplementation was various from 2 to 10 days. However, Luger et al. conducted an intervention to identify the effect of a single dosage of 0.3 g intravenous thiamine supplementation directly before surgery [33]. Two studies reported the serum thiamine status of patients before intervention. In addition, two studies recorded the serum levels of vitamin c in their subjects. After removing retrospective interventions, we enrolled clinical trial articles in the meta-analysis. Characteristics of clinical trial studies have been summarized in Table 2.

**Table 2:** Characteristics of included studies in the meta-analysis

First author (year)	Country	Type of study	Primary diagnosis	Age, year (mean)	Subjects		Vitamin assessment	Intervention		Placebo type	Main outcomes
					Control/ Intervention	Sex (M, %)		Dose (g/day)	Duration (days)		
Iglesias (2020) [23]	USA	RCT	Sepsis	68.5	69/68	45	AA	C1: 6 B1: 0.4 Hydrocortisone: 0.2	4	Normal saline	PE: Vasopressors duration administration Null: – NE: Δ SOFA, mortality, LOS
Chang (2020) [25]	China	RCT	Sepsis	61.65	40/38	54	–	C: 6 B1: 0.4 Hydrocortisone: 0.2	4 4 7	Either normal saline or routine treatment	PE: – Null: Δ SOFA, hypernatremia NE: mortality, ICU LOS, vasopressors duration, length of Mechanical ventilation
Donnino (2016) [31]	USA	RCT	Septic– shock	67	45/43	59	Thiamine	B1: 0.4	7	Normal saline	PE: – Null: – NE: APACHE II, Δ SOFA, LOS, mortality
Blakrishian (2019) [24]	India	RCT	Septic –shock	52	12/12	62	–	C: 6 B1: 0.4 Hydrocortisone: 0.2	4	Normal saline	PE: Dose of noradrenaline and vasopressin Null: – NE: Δ SOFA, mortality
Fujii (2020) [26]	Australia New-Zealand Brazil	RCT	Septic –shock	61.7	104/107	63	–	C: 6 B1: 0.4 Hydrocortisone: 0.2	Up to 10 days	Hydro–cortisone: 0.2	PE: – Null: – NE: Survival, vasopressors-free days, mortality, LOS, Δ SOFA, ventilator free days, ICU free days
Harun (2019) [12]	Malaysia	RCT	Septic -shock	65	33/32	58	–	B1: 0.6	3	Normal saline	PE: – Null: – NE: Vasopressors duration administration, ICU LOS, Δ SOFA, mortality
Hwang (2020) [27]	Korea	RCT	Septic– shock	70	58/53	38	AA	C: Up to 6 B1: 0.4	2	Normal saline	PE: – Null: Δ SOFA, mortality, vasopressor–free days, vasopressor dosage, ventilatorfree days NE: –
Karimpour (2019) [28]	Iran	RCT	Septic- shock	61	50/50	43	–	C: Up to 6 B1: 0.2	4	Normal saline	PE: vasopressors duration Null: Δ SOFA, mortality, ICU LOS, ventilator-free days NE: –
Lomivorotov (2019) [13]	Australia	RCT	Surgery (CABG)	64	20/19	59	–	B1: 0.4	3	Placebo	PE: – Null: vasopressor dose, length of mechanical ventilation, Δ SOFA, LOS, ICU LOS NE: –
Luger (2015) [33]	Australia	RCT	Cardiac- Surgery	58	15/15	77	–	B1: 0.3	Before surgery	Normal saline	PE: concentrations of blood thiamine Null: hospital LOS, 30 days- mortality, ICU LOS NE: –
Moskowitz (2020) [29]	USA	RCT	Septic- shock	68.4	99/101	55	–	C: 6 B1: 0.4 Hydrocortisone: 0.2	4	Normal saline	PE: – Null: Δ SOFA, mortality, ventilator-free days NF: –
Moslemi (2020) [10]	Iran	RCT	Surgery	54	48/48	59	–	B1: 0.2	3	Normal saline	PE: – Null: length of mechanical ventilation, vasopressor use, Δ SOFA NE: –
Nasution (2020) [34]	Indonesia	RCT	Sepsis	46	12/12	–	–	B1: 0.4	3	Normal saline	PE: – Null: Δ SOFA NE: –
Pradita-ukrit (2020) [32]	Thailand	RCT	Cardiac-arrest	64.3	17/20	70	–	B1: 0.3	7	Normal saline	PE: – Null: mortality, ICU LOS NE: –
Wani (2020) [30]	India	RCT	Sepsis	67.5	50/50	59	–	C: 6 B1: 0.4 Hydrocortisone: 0.2	4 4 7	Normal saline	PE: vasopressor duration Null: mortality, length of mechanical ventilation, vasopressor duration, LOS, Δ SOFA NE: –

Outcomes: *NE* Negative Effect, *PE* Positive Effect, *Null* None Effect, *SOFA* Sepsis-Related Organic Failure Assessment, *LOS* length of stay, *AA* ascorbic acid, *CABG* Coronary artery bypass, *ARDS* Acute respiratory distress syndrome

### Thiamine regimens

The results of the studies have been displayed in three main subgroups. The first subgroup was thiamine supplementation which involved seven interventions with thiamine injection [2, 9, 13–16, 22]. The second subgroup included three studies. Intervention for this subgroup was the concurrent injection of thiamine in association with vitamin C [7, 17, 18]. The last subgroup, which included ten studies, was the simultaneous injection of thiamine, vitamin C, and low-dose hydrocortisone (triple therapy) [1, 3–6, 8, 10–12, 23].

### *The effect of thiamine alone, thiamine in association with vitamin C, and HVT on number of ventilator*-*free day*

**Fig. 2:**
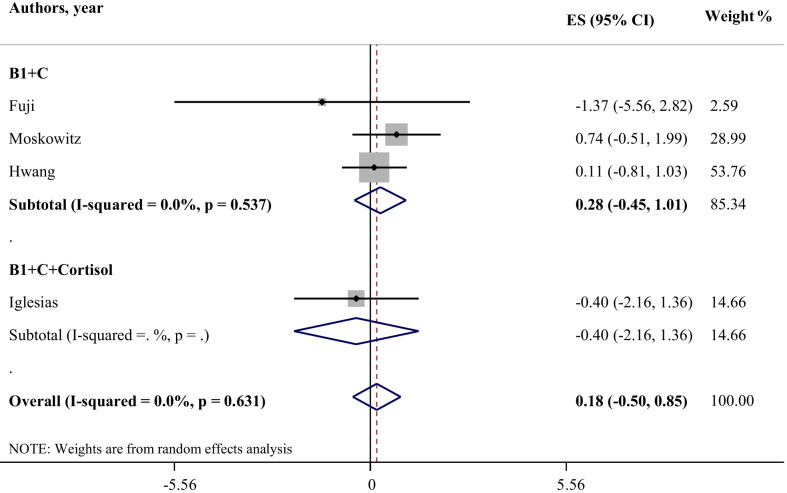
Effect of thiamine alone, thiamine in combination with vitamin C, and HVT (hydrocortisone, vitamin C, and thiamine) on number of ventilation free-day*. *It is assumed that interventions could increase the number of ventilator-free days

The results of present studies revealed no significant effect of thiamine in combination with vitamin C, and HVT on number of free days of ventilation, respectively (WMD: 0.28; 95% CI: − 0.45, 1.01; I-square: 0.0%) and (WMD: − 0.40; 95% CI: − 2.16, 1.36; I-square was not calculated). There were no enough study for statistical tests regarding effect of thiamine alone on number of free day ventilation (Fig. 2).

### The effect of thiamine alone, thiamine in association with vitamin C, and HVT on ICU mortality percentage

**Fig. 3:**
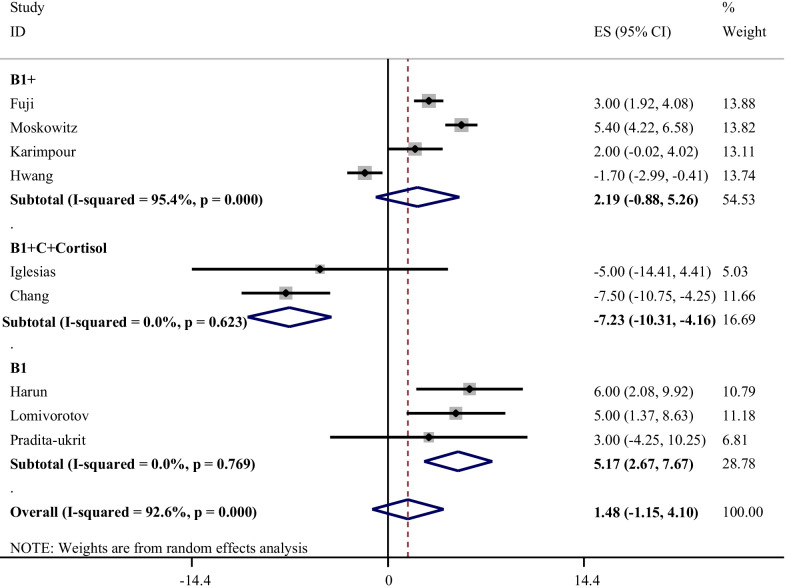
Effect of thiamine alone, thiamine in combination with vitamin C, and HVT (hydrocortisone, vitamin C, and thiamine) on mortality percentage*. *It is assumed that interventions that improve clinical outcomes will associated a reduction in mortality

The effects of different treatments on ICU mortality rate is illustrated in Fig. 3. The results of the meta-analysis of three studies regarding effect of thiamine supplementation on ICU mortality showed that administration of thiamine alone is associated with high mortality percentage without heterogeneity between studies (WMD: 5.17%; 95% CI: 2.67, 7.67; I-square: 0.0%). Thiamine in combination with vitamin C had no significant impact on mortality rate (WMD: 2.19%; 95% CI: − 0.88, 5.26; I-square: 95.4%). In contrast, HVT could decrease mortality rate (WMD: − 7.23%; 95% CI: − 10.31, − 4.16; I-square: 0.0%).

### The effect of thiamine alone, thiamine in association with vitamin C, and HVT on length of ICU stay

**Fig. 4:**
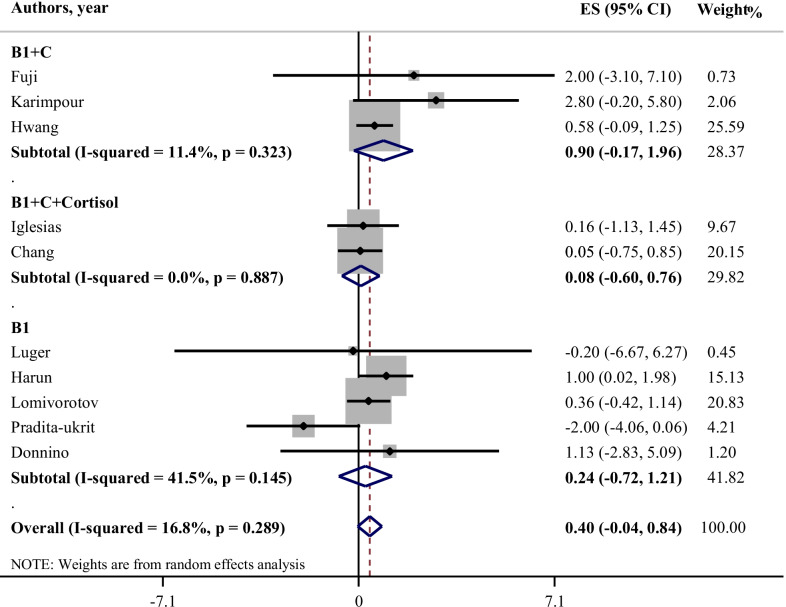
Effect of thiamine alone, thiamine in combination with vitamin C, and HVT (hydrocortisone, vitamin C, and thiamine) on length of ICU stay*. *It is assumed that interventions could reduce the length of ICU stay

The effects of different treatments on length of ICU stay is illustrated in Fig. 4. The meta-analysis of two studies indicated that there was no statistical significant effect of thiamine alone on ICU length of stay (WMD: 0.24; 95% CI: − 0.72, 1.21; I-square: 41.5%). Moreover, supplementation of thiamine in association with vitamin C, and HVT have no significant effect on ICU length of stay (WMD: 0.90; 95% CI: − 0.17, 1.96; I-square: 11.4%) and (WMD: 0.08; 95% CI: − 0.60, 0.76; I-square: 0.0%), respectively**.**

### The effect of thiamine alone, thiamine in association with vitamin C, and HVT on change of SOFA score

**Fig. 5:**
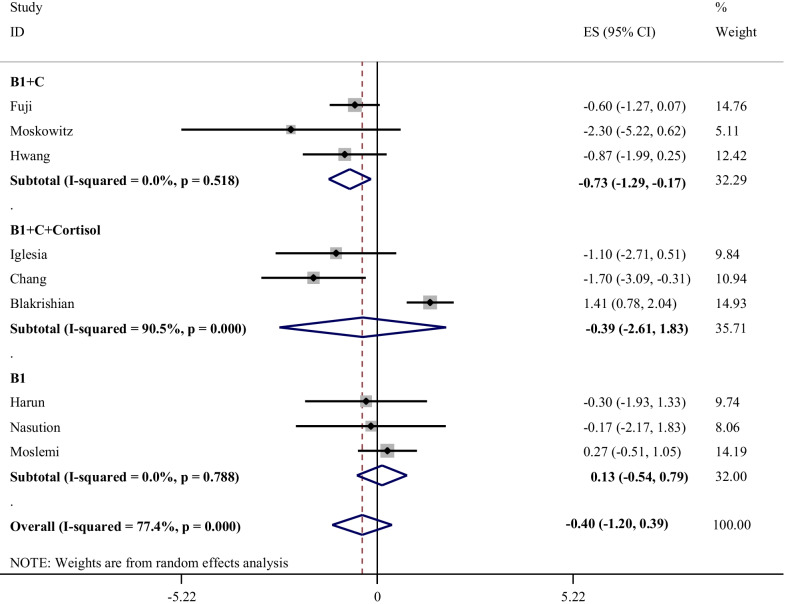
Effect of thiamine alone, thiamine in combination with vitamin C, or HVT (hydrocortisone, vitamin C, and thiamine) on change of SOFA Score*. *It is assumed that interventions that improve clinical outcomes will associate with *decreasing* SOFA score

The effects of different treatments on SOFA score is illustrated in Fig. 5. The results of the meta-analysis showed that thiamine supplementation had no significant effect on SOFA score in the ICU patients (WMD: 0.13; 95% CI: − 0.54, 0.79; I-square: 0.0%). There was no heterogeneity between studies. Interestingly, supplementation with thiamine and vitamin C had a significant decreasing effect on SOFA score (WMD: -0.73; 95% CI: − 1.29, − 0.17; I-square: 0.0%). There was no heterogeneity the between studies**.** Additionally**,** our analysis showed that administration of thiamine, C and low-dose hydrocortisone had no overall effect on SOFA Score (WMD: − 0.39; 95% CI: − 2.61, 1.83; I-square: 95.5%) with high heterogeneity between studies.

### Sensitivity analysis

The sensitivity analysis regarding the effect of thiamine alone on ICU mortality, the number of ventilator-free days, and the SOFA score specified that the results were not impressed by any one study. Concerning the effect of thiamine administration alone on length of ICU stay, after excluding an unmatched study Pradita-Ukrit et al.)[32], heterogeneity between studies was disappeared (WMD: 0.61 [0.01, 1.22]).

Moreover, the results of sensitivity analysis showed that after removing a study conducted by Hwang et al. [27], thiamine supplementation in association with vitamin C has no significant effect on SOFA score (WMD: − 0.83 [− 1.97, 0.31]). In contrast, removing the study by Hwang et al. [27] reduced the heterogeneity among the studies. Therefore, the effect of thiamine in association with vitamin C on length of ICU and ICU mortality becomes significant ((WMD: 2.59 [0.01, 5.18]), (WMD: 3.57 [1.62, 5.51])), respectively. There were no evidences of publication bias for studies regarding effect of thiamine supplementation in combination with vitamin C on number of ventilator-free days.

The sensitivity analysis concerning effect of HVT on number of ventilator free days, and length of ICU stay also revealed no evidences of heterogeneity to be introduced. However, through sensitivity analysis, we found that results of HVT on ICU mortality, and changes of SOFA score was affected by Chang et al. [25], and Balakrishnan et al. [24], respectively [(WMD: − 5 [− 14.41, 4.41]), (WMD: -1.44 [− 2.49, − 0.39])].

## Discussion

### The effect of thiamine alone on clinical outcomes in ICU patients

In the current meta-analysis, we identified that administration of thiamine alone had no significant effect on SOFA score, and number of ventilator-free days in ICU patients. In contrast, thiamine supplementation increased mortality percentage in ICU.

Thiamine is an essential cofactor for the synthesis of ATP in mitochondrial and function of antioxidant enzymes, as well as it has the critical role in neurological and cardiac function [35, 36]. The hypermetabolic state, administered loop diuretics, and intensive care treatments can lead to thiamine deficiency in critically adult patients [36, 37]. It seems that the ameliorating effect of thiamine on end-organ damage could be hypothetically associated with the cells' adenosine triphosphate increasing and reduction in lactic acid production, and consequently might have a protective effect on mortality [38]. Unexpectedly, in the current analysis, the increased mortality caused by thiamine administration may only be due to the following reasons. First, it has been previously documented that thiamine administration has no significant effect on lactate clearance in extensive surgery patients [9]. Second, we could not enter some stated positive results related to thiamine treatment on mortality of critically ill patients with septic shock into our analysis, due to inappropriate reported data [39]. Another possible reasons may be related to the status of thiamine deficiency at baseline of intervention, and the median time from admission to thiamine administration in critically ill patients that unfortunately has been not clarified across the included studies [12, 17]. It also should be considered that, besides the thiamine deficiency, serum lactate levels could be affected by other factors, including anaerobic glycolysis due to hypoperfusion and microcirculation abnormalities, and impaired hepatic lactate clearance [40, 41]. Consistent with our study, the findings of a nationwide database-based observational study concerning exanimating the effect of thiamine administration on mortality in 68,571 eligible patients indicated no support for thiamine administration early after admission and the 28-day mortality in patients with septic shock [42]. Moreover, a recent review study by evaluating 122 papers found that although thiamine deficiency may be more prevalent in patients admitted to ICU, there is a lack of consensus on the benefits of thiamine supplementation in the main outcomes of patients [43]. Therefore, we hypothesized that the positive effect of treatment with thiamine alone on clinical outcomes in critically ill patients could be affected by several factors, including organ function score, the time of thiamine prescription, thiamine deficiency, and lactic acidosis status, particularly in cases with identified nutritional risk factors. Consequently, these results could not be generalized to patients with other conditions, and the potential therapeutic impact of thiamine in the overall ICU population remains unknown.

### The effect of thiamine in combination with vitamin C on clinical outcomes in ICU patients

Our pooled analysis showed that the combination therapy with thiamine and vitamin C had no significant impact on ICU mortality, number of ventilator free days, and ICU stay. Treatment with thiamine in combination with vitamin C could decrease SOFA score in critically ill patients with sepsis. However, sensitivity analysis revealed that after removing an unmatched study, the mentioned effect on SOFA score has disappeared.

There is evidence that vitamin C levels can decline dramatically as a result of acute inflammation of many physiological stress conditions, such as sepsis, traumas, extensive surgery, and burns [44–46]. As an antioxidant, vitamin C has an important function in cell protection against inflammation and oxidative stress-induced cellular damage [47]. Providing data on circulating antioxidant levels in the largest cohort of septic patients also showed that the capacity of antioxidant serum levels was associated with 30-day survival (hazard ratio = 1.50, 95% confidence interval = 1.16–1.94, *P* = 0.002) [48]. In this concern, many studies have examined the potential effect of vitamin C and thiamine on the SOFA score, as the prognostic indicator of mortality in patients with sepsis and post-cardiac arrest syndrome [49, 50]. In confirmation with our study, Soto et al. also revealed the benefits of antioxidant therapy on multiple organ dysfunction in patients with Coronavirus infection [51]. In contrast, recent published systematic review and meta-analysis concerning impact of vitamin C on patients with sepsis reported that vitamin C supplementation did not have any effect on survival and ICU stay in critically ill patients [52]. However, there are some limitations in mentioned study. First, the information of studies on vitamin C administration were pooled and analyzed in combination with thiamine to examine the effect of vitamin C on ICU patients. In addition, the majority of included studies had retrospective design.

Therefore, we assumed that combination therapy with thiamine and vitamin C might enhance organ function by cell damage reduction, modulation of inflammatory status, and the neurological protection in organs. However, the differences in inflammation status and antioxidant levels of participants could determine other benefits of this combination therapy on ICU outcomes [53]. On the other hand, we showed that improved SOFA score did not lead to a mortality benefit in patients who received thiamine and vitamin C combination therapy. Although the change in SOFA score has been considered as the primary outcome along with reporting mortality in several studies, some points should be attended in the interpretation of Delta Sofa (ΔSOFA) score in our study. According our sensitivity analysis, after removing an unmatched study conducted by Hwang et al. [27], the significant impact of thiamine and vitamin C combination therapy on SOFA score has disappeared. Since the characteristics of study population in mentioned study, including vitamin C deficiency (approximately 50% of participants) and gender (female, 62%) might affect the results of our analysis, these statistically significant changes in SOFA score should be cautiously interpreted. Moreover, this outcome was evaluate only in patients who were alive in the ICU on 72-h, that means data regarding participants who early discharge from the ICU due to recovery or death were not considered in this analysis. Moreover, through sensitivity analysis we found that our pooled analysis of the changes of SOFA score was affected by Balakrishnan et al. study [24]. For this reason and because of the mentioned variety for measurements and patients' conditions, our finding should be interpreted with caution. Consequently, recommended thiamine and vitamin C dosage may vary according to individual requirements and may need to administrate based on the patients' conditions individually.

### The effect of HVT on clinical outcomes in ICU patients

In the present study, we showed that the HVT protocol could decrease mortality rate. In contrast, HVT had no significant effect on other clinical outcomes.

In the early stage of sepsis, acute organ dysfunction caused by the release of numerous cytokines and metabolic abnormalities emphasizes the important role of early treatment in restoring vascular endothelial function and the reduction of inflammation in these patients [54]. Some previous studies documented that the early treatment with low-dose hydrocortisone in combination with thiamine and vitamin C could improve organ function and prevent organ injury in patients with septic shock, as indicated by the reduction in SOFA scores [55, 56]. The findings of recent review also showed that administration of low-dose hydrocortisone, ascorbic acid and thiamine (HAT therapy) may reduce organ dysfunction in patients with sepsis and improved mortality outcomes [57]. Moreover, a recently published meta-analysis by Shi et al., on overall results from cohorts revealed that HVT could significantly reduce mortality (RR0.46, 95% CI 0.25 to 0.86, *p* = 0.01; *I*^2^ = 75%, *P*_*H*_ = 0.001), but not the duration of vasopressors use (WMD 1.11 h, 95% CI − 59.60 to 61.82, *p* = 0.97; *I*^2^ = 98%, *P*_*H*_ < 0.001) [58]. However, low dose corticosteroids administration could not effect on short- and longer term mortality in adult's patients with septic shock [59].

Clinically, the correlation between adverse outcomes of respiratory infections and hypertension was indicated and attributed to increased expression of Angiotensin-converting enzyme II (ACE2) [60]. Recently, it has been shown that ascorbic acid administration can play controlling role on cellular expression of ACE2 [61]. On the other hand, administration of low-dose hydrocortisone is recognized as the strongest activator of ACE2 [62]. In addition, the different effects of hydrocortisone on organ function were reported based on dose and time of administration. [63]. It seems that the addition of thiamine and vitamin C, as antioxidants, modulate the effects of hydrocortisone therapy, and may facilitate the binding of glucocorticoids to their receptor [64]. Moreover, impairment of glycemic control, as an important side effect during steroid administration may moderate through regulation of glucose metabolism by thiamine therapy [65]. These results confirm the differences in the effects of intervention between patients at the different stages of sepsis and primary diagnosis condition.

However, in contrast to positive effect of thiamine and vitamin C supplementation on the SOFA score, HVT therapy had no significant improvement in the change of SOFA score in our study. One of the possible reason for this result might be that the SOFA scores calculated at different intervals times in three included studies, SOFA measurement intervals at first 72 h [23, 25] versus 96 h [24]. In addition, the type of disease diagnosed in hospitalized patients, including sepsis and surgery, might make a difference in the outcomes. The recent results of treatment with vitamin C, thiamine, and hydrocortisone indicated a significant reduction in the SOFA score in the intervention group in comparison to the control group in patients with sepsis [68]. Therefore, we speculate that the results of this combination treatment through antioxidant, anti-inflammatory, and immune-enhancing properties depend on patients' conditions. The lack of adequate data in this area suggests more randomized clinical trials regarding the effects of combination treatment on clinical outcomes in ICU patients with various primary conditions, such as surgery or trauma.

### Limitations and strengths

Recently, several meta-analysis studies determined the effect of vitamin C with or without thiamine treatment and reassessed the value of HVT administration in patients with sepsis [66–68]. Strengths of the current systematic review, including the use of a comprehensive database evaluating and consideration of several recently published studies, and the examination of the value of thiamine administration in three main subgroups (thiamine administration individually, thiamin in combination with vitamin C, and HVT through 15 clinical studies that can improve our information concerning the management of clinical outcomes in critically ill patients. This meta-analysis, nonetheless, has potential limitations that should be considered. First, as stated, the pooled effect of thiamine alone or in combination with vitamin C was evaluated only on patients with sepsis or under surgery due to data deficiencies. Second, since the results related to mortality in the eligible studies was reported as percent changes, we typically could not identify risk difference. In addition, due to the limited reporting on the topic of impact of thiamine administration alone and HVT, we consequently could not analyze and determine the effect of thiamine administration on number of ventilation free day. In addition, because of limited data, we could not evaluate the heterogeneity regarding the large variability of SOFA measurement intervals by subgroup analysis. The heterogeneity of background conditions of patients from sepsis to trauma also is another limitation highlighting the need for more randomized clinical trials regarding the effects of combination treatment on clinical outcomes with various primary conditions of patients admitted to ICU.

## Conclusion

In contrast to HVT, thiamine supplementation was associated with increased mortality rate in ICU. In addition, co-supplementation of thiamine and vitamin C had a significant decreasing effect on SOFA score.

## Data Availability

Not applicable.
